# Dr. Rustum Roy (1924–2010)

**Published:** 2010

**Authors:** Dilip Mehta

**Affiliations:** *Viridis Biopharma Ltd., Mumbai E-mail: dilipmehta@gmail.com*

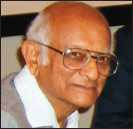



Dr. Rustum Roy, one of India’s most distinguished scientists of recent decades, whose article, “Integrative medicine to tackle the problem of chronic disease,” was published in *J-AIM* 1(1), passed away on 26th August 2010 at the age of 86. Active till the end, Roy had been a pioneering materials scientist, a proponent of holistic and integrative medicine, and advisor and critic of U.S. science policy. For the last 60 years this son of India had been on the faculty of Pennsylvania State University (PSU), where he was one of the university’s greatest ever scientists. He held the Evan Pugh Chair of Solid State as one of five professorships in different fields at different universities.

Born in 1924, Roy received B.Sc and M.Sc degrees from Patna University and his PhD from PSU. He spent 65 years there, first as a graduate student, and then faculty member, founding its Materials Research lab in 1962. The lab was later ranked first in the world by the Institute for Scientific Information on the basis of the number of its highly cited scientists.

Most remarkable was Roy’s breadth of knowledge and interest outside material science, ranging *inter alia* from science policy, radioactive waste management, sexual ethics, science of religion, and, importantly, path-breaking contributions to whole person healing (alternative medicine). His recognition in other fields set him apart from his science and engineering colleagues. He was chosen to give the “Hibbert lectures,” a prestigious lecture series on religion and philosophy in the UK; he was the chair of “Friends of Health,” which examines disruptive effects on human health due to innovations in materials science and physics rather than biochemistry. His five professorships included “Visiting Professor of Medicine” at the University of Arizona. He was as much recognized for his contributions to medicine and human healing as for material science and physics.

Rustum Roy was the first Indian elected to the US National Academy of Engineering, as also to the National Academies of Science and Engineering of Japan, Switzerland, India, and Russia, a rare and distinctive achievement. As one of today’s most prominent scientists, he also won innumerable national and international awards, including Japan’s equivalent of a “knighthood.” As much at home among the world’s leading artists, clergy, theologians, and healing gurus as among his fellow scientists and engineers, he will long be remembered for championing interdisciplinary and integrative learning; he was so special on many levels. Incredibly brilliant, he contributed to many fields, opening unique doors. He understood; he will truly be missed. Although no longer present, the mission and vision of this brilliant scientist and humanitarian will continue to bear fruits through his students and followers.

Roy is survived by his wife Della Martin, herself a reputed material scientist, and their three children.

